# Optimizing data transmission in 6G software defined networks using deep reinforcement learning for next generation of virtual environments

**DOI:** 10.1038/s41598-024-75575-y

**Published:** 2024-10-28

**Authors:** Khaled Mohamed Naguib, Ibrahim Ismail Ibrahim, Mahmoud Mohamed Elmessalawy, Ahmed Mostafa Abdelhaleem

**Affiliations:** 1 CCAS Department, School of Engineering, New Giza University (NGU), Giza, Egypt; 2 Department of Electronics and Communications, Faculty of Engineering, Helwan University, Cairo, Egypt

**Keywords:** 6G cellular networks, Virtual reality, Software defined network, Deep reinforcement learning, Network slicing, Latency, Achievable data rate, Electrical and electronic engineering, Computational science, Computer science, Software

## Abstract

Data transmission of Virtual Reality (VR) plays an important role in delivering a powerful VR experience. This increasing demand on both high bandwidth and low latency. 6G emerging technologies like Software Defined Network (SDN) and resource slicing are acting as promising technologies for addressing the transmission requirements of VR users. Efficient resource management becomes dominant to ensure a satisfactory user experience. The integration of Deep Reinforcement Learning (DRL) allows for dynamic network resource balancing, minimizing communication latency and maximizing data transmission rates wirelessly. Employing slicing techniques further aids in managing distributed resources across the network for different services as enhanced Mobile Broadband (eMBB) and Ultra-Reliable and Low Latency Communications (URLLC). The proposed VR-based SDN system model for 6G cellular networks facilitates centralized administration of resources, enhancing communication between VR users. This innovative solution seeks to contribute to the effective and streamlined resource management essential for VR video transmission in 6G cellular networks. The utilization of Deep Reinforcement Learning (DRL) approaches, is presented as an alternative solution, showcasing significant performance and feature distinctions through comparative results. Our results show that implementing strategies based on DRL leads to a considerable improvement in the resource management process as well as in the achievable data rate and a reduction in the necessary latency in dynamic and large scale networks.

## Introduction

The upcoming sixth generation (6G) mobile network is promising to deliver data fast with super-efficient transmission to keep dealing with new technologies. One major focus of 6G is improving user application experiences like Virtual Reality (VR) and Augmented Reality (AR) which using these technologies will become easier wherever you are without any restrictions. This support is expected to super huge growth of VR/AR services and apps whereas in turn this will push forward the incredible development of VR/AR gadgets. In this context, the way of interacting with media will become for users by super-realistic AR/VR immersive experiences and even possible using holographic interactions become a commonplace. VR/AR are technologies to become a key essential part of 6G but increase incredible demand for high data rates and very low latency, which will push the limits of 6G networks. To meet these demands, 6G networks will need to ensure VR users have the necessary resources, like computing power and storage, which could involve leveraging a vast network of VR^1^. In the realm of 5G mobile networks, there are three mainly communication scenarios: enhanced Mobile Broadband (eMBB), massive Machine-Type Communications (mMTCs), and Ultra-Reliable and Low Latency Communications (URLLCs). Concurrently, both academia and industry have initiated research into the realm beyond 5G (B5G) and 6G. 6G networks will introduce more vertical application scenarios with specialized capabilities, such as latency and peak data rate, projected to emerge beyond 2030. These scenarios are not limited to the three major ones in 5G whereas 6G networks are anticipated to possess robust capabilities for delivering tailored and adaptable services. Network Slicing (NS) is widely acknowledged as a fundamental facilitator of 6G networks. Through slicing, the network functions and resources of all physical elements can be abstracted comprehensively by enabling straightforward management, allocation, and deployment^2,3^.

In the same manner, advancements in softwarization and virtualization technologies illustrated by software-defined networking (SDN) and network function virtualization (NFV) are advancing the progression towards 6G NS. Despite the promising potential of 6G NS, several technical challenges arises through its seamless implementation. One critical challenge is resource allocation in 6G NS among different slices. Consequently, resource allocation 6G NS emerges as a pivotal technical concern deserving further research emphasis. Within this framework, the sophisticated task of mapping sliced end-to-end services onto the underlying 6G networks is raised with extensive access, transport, and core components. Simultaneously, the resource and functional requirements of customized slices must be sufficiently met^4^. Significant efforts has been drawn in the direction of how to better NS performance explained. Usually, optimization techniques used for formulating network resource slicing problem. Nevertheless, this difficult problem of NS is fraught by the fact that Radio Access Network (RAN) environment is very dynamic. In doing so, 6G NS must embrace more intelligent resource allocation capabilities to ensure low latencies and increased data rates for all users. To this end, harnessing machine learning/artificial intelligence capabilities such as Deep Reinforcement Learning (DRL) algorithms may offer promising solutions^5^.

## Literature review

In recent research, the domain of virtual environment communications has encountered a surplus of challenges which is driven by escalating the needs for advanced and more effective communication systems. To tackle these problems, massive research has been undertaken which focuses on the capabilities of 6G NS and SDN within this domain. Many of the following researches used Deep Reinforcement Learning (DRL) as solvent for optimizing network problems. Virtual environment applications are tackled by high data rates and low latency which 6G can offer these requirements. This article^6^ suggests using modern machine learning techniques such as multi-armed bandits and federated learning to help solve problems in metaverse development which is one of the virtual environments. This includes optimizing resources and improving how users experience satisfied. Two examples are efficiently allocating VR resources and customizing content for each user’s interests and needs. In^7^, it found that Rate Splitting Multiple Access couldn’t meet the demands needed for virtual reality streaming in 6G networks due to computational and dynamic user demand challenges. The authors found a solution that came up with a new method which is a special mix of multicast and deep reinforcement learning, thus, the system meet the strict timing needs and rates required. This study^8^ introduced a new way to schedule tasks for wireless virtual reality in 6G networks. It uses cooperation between edge servers to make better the task distribution. The authors used a method where no central control is needed and multiple agents work to optimize tasks that hac to get offloaded successfully with less delay and balanced loads.

SDN are used to centralized management through 6G cellular networks. In^9^, authors studied how Machine-to-Machine (M2M) is changing, especially in Internet of Things (IoT) surroundings. They highlighted 6G role in meeting IoT future needs which they explained a scalable SDN in-band control protocol, using a data network for controlling information transmission made for IoT networks. Carried out in wired and wireless networks with Basic OpenFlow User-space Software Switch (BOFUSS), network flexibility and programmability was improved which is important for better IoT performance in 6G. In another article^10^, the authors presented design for 6G networks using SDN as the main link for continuous network checks and transmission links selections. They used SDN for tidy resource distribution and keeping an eye on data flow which manages contradicting performance measures with a DRL guided algorithm and Soft Actor-Critic (SAC) technique. NS is a promising technique in 6G with more flexible technologies. This study^11^ investigated a new way to share network resources in 6G systems which solve the tough task of managing different types of wireless communication on limited radio waves. By using cognitive radio and slicing network, the study built a model to suggest an idea for sharing the radio spectrum which can improve usage of network channels. This research^12^ tackles 6G IoT apps by blending Joint Connection And Sensing (JCAS) with complete NS. It used deep reward focused learning to properly allocate resources and cut full system lag time. The simulated results prove its ability to meet service quality standards and the needs of future wireless networks. Solving the problem of sharing messages in IoT for social situations studied in^13^. This study showed way to use Unmanned Aircraft Systems (UAS) and fog computing. It uses a system of layers to send messages and takes advantage of the best routes using a 6G network slice using SDN. This makes messages sent to their destination faster and covers a wider area, while also making sure achievement of less delay.

Radio Access Network (RAN) slicing is the most critical aspect to a successful network in this sense and its optimization entails a consideration of parameters and techniques such as Dynamic Spectrum Sharing (DSS), load balancing, and interference management. Such basic management strategies as QoS management and admission control are vital for fulfilling Service Level Agrement (SLA) demands along with having a minimal negative effect on other slices. RAN slicing is categorized based on resource allocation methods: In Static Slicing the resources are held constant once after the network planning stage in order to provide definite QoS, but in Dynamic Slicing the resources are changed quite often according to the traffic and hence it can be effectively implemented for applications which show variation in traffic. BS-Level Slicing divides the resources for wireless along the different base stations, while Network-Level Slicing uses a controller to manage the resources of different BSs thereby offering more chances of network customization^14^. The optimization of resource allocation in RAN slicing to deal with the different QoS requirements and can serve users of the Mobile Virtual Network Operator (MVNO); to deal with this, this work presents the Modulation and Coding Scheme (MCS)-aware RAN slicing problem as a Non-Linear Programming problem and proposes a greedy algorithm to solve it and it is contrasted with an upper mechanism bound to support minimum data rates for all users^15^. In^16^, an improved network slicing framework is proposed for 5G and beyond networks to optimize the placement of NFVs, radio access points, data flows and compression to achieve different Key Performance Indicators (KPIs) related to reliability, energy consumption, data quality while improving the existing approaches mainly associated with throughput and delay. In^3^, a novel network architecture for 6G Fog Radio Access Networks (F-RAN) that optimizes energy efficiency and delay using virtual slicing (VS), and introduces a low-complexity EE optimization algorithm that improves energy efficiency by 30% while maintaining a 1 ms delay threshold. In^17^, 5G RAN slicing in the next-generation networks was discussed based on the theoretical framework with NFV mapping algorithms and slice recovery optimization. To enrich NFV mapping and resource allocation, the authors presented four algorithms, namely Resource-based (RBA), Connectivity-based (CBA), Group-based (GBA), and Group-Connectivity-based (GCBA); extensive experimental results demonstrate that the proposed algorithms achieve excellent performance in the construction of stable RAN slicing. This paper focuses on the adaptation of Edge Computing (EC) with NS through improving the dynamic slice scaling and task offloading of the involved service providers in 5G networks. The authors in^18^ involved a low-complexity algorithm to offload tasks/modules and allocate radio and computing resources in real-time, and a Deep Q-Learning (DQL) framework for reconfiguration. The network slicing framework is experimentally evaluated by using a network slicing testbed with Docker and with real traffic trace and a sample augmented reality application to optimize the real-time offloading ratio and resource allocation policy of slice requests.

SDN can be a technique that realises virtualization which is associated with resource slicing or the process of slicing up physical network infrastructure and spectrum based resources into multiple NFV. As a result, a number of applications can be controlled by dividing them by the necessary bandwidth and data storage onsite, while using a single network infrastructure. This process is improved in SDN, particularly, by the utilization of a centralized controller that determines the allocation of resources depending on collective QoS demands and current status of the channels and improves the effectiveness and adaptability of the slicing of the network. To meet these challenges and requirments, this paper presents a model of SDN system consisting of VR that is intended to address the transmission requirement of the VR video in 6G cellular networks. The centralization of SDN helps in network resource managing and NS throughout communication with VR users and base stations and supports both low-latency and high-data rate communication for efficient resource use. The proposed approach here is to use DRL for selecting the resources, calculating the weighting factors, and forming perfect NS. Key contributions include:

- A new architecture of an effective VR data transmission management in 6G network with Virtual Reality SDN system model for end-to-end (E2E) VR link between users.
- An optimization problem within the entities to achieve maximum attainable data rates given limited E2E latency and limited resources in each slice focusing on the VR slice requirments.
- As a solution to the VR data transmission needs, the utility of DRL tactics has been demonstrated.
- Performance and Functional Comparisons between different DRL models are demonstrated.

The paper is structured as follows: firstly, an overview of the system model is provided, then, the problem definition is proposed. Thereafter, DRL technique for resource selection optimization with end-to-end management is demonstrated, moreover, the results and discussion are showed, and lastly, the conclusion is written to show all analysis of our proposed model.

### System model

The third Generation Partnership Project (3GPP) has a provision for the slice designs which is an important stage in RAN slicing and precedes the actual slice creation process during planning^19^. The slice design plays a vital role, aiming to meet the communication needs of the services that each slice is intended to support. In many traditions, slices have been distinguished based on radio resource assignments that must satisfy bandwidth or data rate demands. However, this approach does not address the issue of latency requirements, in particular those which are crucial when it comes to 5G powered verticals such as Industry 4.0. This study recognizes and addresses this drawback by recommending new definitions of RAN slices which consider more than just bandwidth or rate demands when considering communication needs.

**Figure 1:**
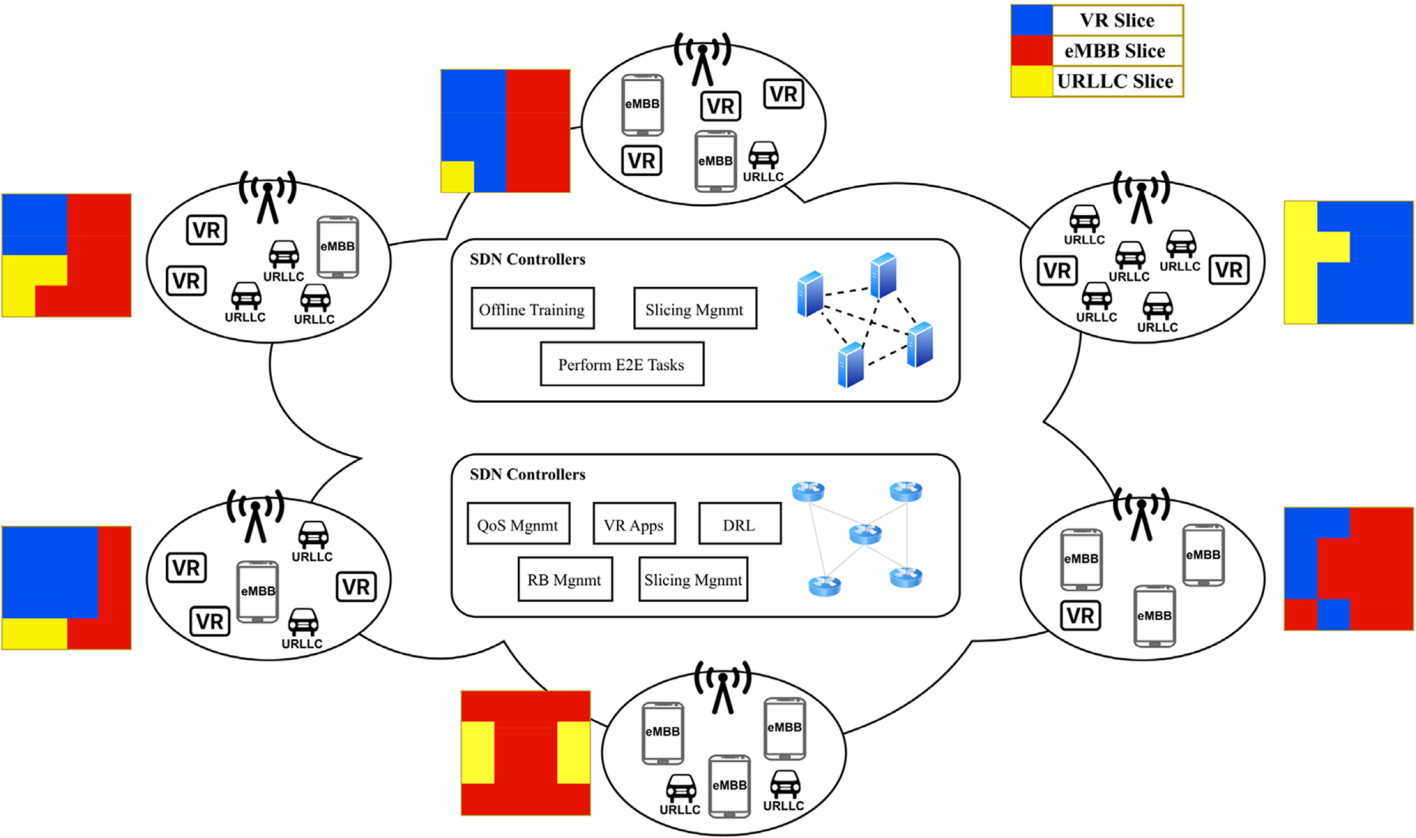
System model.

The scenario proposed in this paper is Orthogonal Frequency Division Multiple Access (OFDMA) within an N-cellular network Base Stations (BSs). In Fig. 1, each BS is positioned at the center of its respective cell and M user equipment (UEs) are uniformly distributed over the network area. These distributed UEs with various service types such as eMBB, URLLC, and VR slices, show how diversified services within this network could be. The recommended VR slice aims at supporting high data rates with low delay. In this network, URLLC services feature short packets while eMBB services are characterized by constant traffic having large packet sizes. Additionally, those UEs that offer VR service support large packets which means that distribution of these large packets across the network’s resources must be done efficiently through network resource management to satisfy strict low-latency demand.

The radio interface between BSs and UEs is structured into frames, with every frame having time and frequency partitions. Frames are split into Transmission Time Intervals (TTIs) or subframes in the time domain whereas bandwidth is divided into subcarriers in the frequency domain. The physical layer of 5G Radio Access Networks (RANs) offers a lot of flexibility to waveform utilization and time-frequency frame structures hence making possible configurations of diverse TTI duration and subcarrier spacing called numerologies. The selection of numerology depends on the deployment frequency band and service requirements for the slice^20^. For this matter, we assume a TTI length of 0.5 ms and sub-carrier spacing of 30 MHz. An essential point to note is that different network slices can exist within the same radio frame, which employ different numerologies. Each network slice is allocated a small time-frequency resource in the radio interface, where the smallest subdivision is called Resource Block (RB) Resource block contains 1 TTI and 12 subcarriers, which in our typical scheme within of 1 millisecond × 180 MHz They are equivalent. Each slice executes its own scheduler, which is responsible for distributing its resource blocks among its users on each TTI, corresponding to the type of distributed service type (eMBB, mMTC, or VR). The number of resource blocks assigned to each slice can be adjusted periodically known as a decision step or steps, usually spaced by several frames In each step, the control agent selects a control action, and it only determines the number of resource blocks available for each slice until the next decision step In between, the control agent collects each slice measurement of user data traffic, channel quality conditions, and Service Level Agreement (SLA) compliance parameters.

The physical network is partitioned into N segments, with each RB potentially allocated to either eMBB, URLLC, or VR network slice. These sets of slices for eMBB, URLLC, and mMTC are represented as I, J, and L, respectively. The total count of eMBB, URLLC, and VR network slices corresponds to I, J, and L, respectively, with the constraint that their sum equals $\;{\text{N}}_{s}$ (I + J + L = $\;{\text{N}}_{s}$). Additionally, $\;{\alpha\;}_{e,i}$, $\;{\alpha\;}_{l,i}$ and $\;{\alpha\;}_{v,i}$ are indicators that RB is allocated for eMBB, URLLC and mMTC, respectively and shown as following:

1 $$\;{\alpha\;}_{e,i}=\left\{\begin{array}{c}1,\;\;\;ifRB\;is\;allocated\;to\;\\\;\;\;\;\;\;\;\;\;\;\;i-th\;eMBB\;slice\;user\\\;0,\;\;\;\;\;\;\;\;\;\;\;\;\;\;\;\;\;\;\;\;\;\;\;other\;wise\end{array}\right. ,\;{\alpha\;}_{l,i}=\left\{\begin{array}{c}1,\;\;\;ifRB\;is\;allocated\;to\;\\\;\;\;\;\;\;\;\;\;\;\;\;\;i-th\;URLLC\;slice\;user\\\;0,\;\;\;\;\;\;\;\;\;\;\;\;\;\;\;\;\;\;\;\;\;other\;wise\end{array}\right. ,\;{\alpha\;}_{v,i}=\left\{\begin{array}{c}1,\;\;\;ifRB\;is\;allocated\;to\;\\\;\;\;\;\;\;i-th\;VR\;slice\;user\\\;0,\;\;\;\;\;\;\;\;\;\;\;\;\;\;\;\;\;\;\;\;other\;wise\end{array}\right.$$

In SDN network used, the control plane is responsible for controlling the network through the northbound interface of SDN. A standard controller is used to centralize global network resource administration and OpenFlow (OF) protocol is employed to communicate with switches and obtain network information, resulting in efficient network resource management and optimization. In SDN, a set of controllers $\;\mathbb{C}=\{\text{1,2},\dots\;\;,C\}$ are considered physically distributed but logically centralized and connected south to OF switches mentioned as set $\;\mathbb{I}=\{\text{1,2},\dots\;,\;I\}$. The importance of SDN is to control the whole link for VR users in End-to-End (E2E) manner. Assume SDN switch $\;i\in\;I$ either managed alone or integrated with BS $\;n\in\;N$. Each BS has a set of VR devices $\;\{{M}_{n}:\;{M}_{n}\subseteq\;M,\;{\sum\;}_{\forall\;n}{M}_{n}=M,\;n=\text{1,2},\dots\;\;,\;N\}$. Let the set $\;\mathfrak{R}=(1,\;\dots\;\;,\;R)$ denote the subcarriers (i.e. RBs) assigned to each BS.

For each VR user $\;{m}_{n}\in\;{M}_{n}$ in BS n, data rate for user $\;{m}_{n}$ in uplink which is denoted by $\;{r}_{m,ul}$ and data rate for user $\;{m}_{n}$ in downlink is denoted by $\;{r}_{m,dl}$. They can be expressed by:

2 $$\;{r}_{m,ul}=W.{\text{log}}_{2}\left(1+\frac{{p}_{n,ul}\left|{h}_{mn}\right|{{d}_{mn}}^{-\zeta\;}}{{\sigma\;}^{2}}\right)$$

3 $$\;{r}_{m,dl}=W.{\text{log}}_{2}\left(1+\frac{{p}_{n,dl}\left|{h}_{mn}\right|{{d}_{mn}}^{-\zeta\;}}{{\sigma\;}^{2}}\right)$$

Where $\;{h}_{mn}$ is the channel information for the link between VR $\;{m}_{n}$ associated to BS n,$\;\;{p}_{n,ul}$, $\;{p}_{n,dl}$ is the transmitting uplink and downlink powers, respectively, W represents the bandwidth of each uplink or downlink subcarrier across all channels, $\;{d}_{mn}$ is the distance between VR user $\;{m}_{n}$ and BS n and $\;{\sigma\;}^{2}$ is the variance of the Additive Gaussian White Noise (AWGN). VR user data sent to its associated BS is determined by $\;{S}_{m,ul}$. Data can also be received from a virtual server in controller c or from other users within the same or different BSs, this data is measured by $\;{S}_{m,dl}$. The link between two VR users is denoted as $\;{l}_{i,j}$. A communication scenario is considered for each VR user in every link such that large data amounts need to be sent with minimum latency and with high data rates. The latencies for transmission and receiving are jointly managed and fluctuate depending on the data volume being transmitted or received. This ensures that the E2E latency remains within the minimum threshold for optimal VR performance. The necessary latency to enhance the performance of VR users, whether it’s transmitting its interaction with the environment or receiving real-time changes in other VR users environment interactions, is defined as:

4 $$\;{L}_{m,ul}=\frac{{S}_{m,ul}}{{r}_{m,ul}}$$

5 $$\;{L}_{m,dl}=\frac{{S}_{m,dl}\;}{{r}_{m,dl}}$$

SDN Network assigns uplink or downlink rates to VR users based on whether they’re transmitting or receiving in a given time slot. Whereas, $\;{L}_{J}$ denotes the time interval for a network slice request in the backhaul. The duration to process data sent to switch *i* is represented by $\;{L}_{i}$​. Upon reception in the backhaul controller, data processing time is $\;{L}_{c}$​ to be handled within its corresponding tenant application. Packets sent originated from or requested by VR users in another BS n, or they might be served from a virtual environment hosted on a specific server within controller c. From previous analysis the time required within the SDN network for serving packets is defined as:

6 $$\;{L}_{SDN}={L}_{J}+{L}_{i}+{L}_{c}$$

### Problem formulation

The main objective is to maximize the achievable rate during communication among VR users or interaction with the environment. However, achieving minimum E2E latency is crucial for latency-sensitive VR applications. While the rate in uplink and downlink may vary based on the data size transmitted, the latency differs while transmitting data from a VR user to a BS or on reverse. Consequently, the E2E time for the link $\;{l}_{i,j}$​ can be described as:

7 $$\;{TL}_{i,j}=\beta\;{L}_{m,ul}+{L}_{SDN}+\left(1-\beta\;\right){L}_{m,dl}$$

Where, $\;\beta\;$ is a weighting parameter between uplink and downlink latency to satisfy transmitter and receiver Quality of Service (QoS) as mentioned in both scenarios. Thus, the overall system data rate can be calculated as:

8 $$\begin{aligned} \;{R}_{total}=&\sum\;_{n=1}^{N}\Bigg[\sum\;_{j=1}^{{U}_{n,VR}}\sum\;_{\begin{array}{c}i=1\\\;i\ne\;j\end{array}}^{{U}_{n,VR}}\;({\alpha\;}_{v,i}{a}_{j,n}{r}_{j,n}^{dl}+{\alpha\;}_{v,i}{a}_{i,n}{r}_{i,n}^{ul}) \\&+\sum \;_{i=1}^{{U}_{n,eMBB}}({\alpha\;}_{e,i}{a}_{i,n}{r}_{i,n}^{dl}+{\alpha\;}_{e,i}{a}_{i,n}{r}_{i,n}^{ul})+\sum\;_{i=1}^{{U}_{n,uRLLC}}({\alpha\;}_{l,i}{a}_{i,n}{r}_{i,n}^{dl}+{\alpha\;}_{l,i}{a}_{i,n}{r}_{i,n}^{ul})\Bigg] \end{aligned}$$

Where $\;{a}_{i,n}$ and $\;{a}_{j,n}$ are indicators of VR user $\;{m}_{n}$ associated to BS n in uplink and downlink, respectively. Thus, the optimization problem is formulated as:

9 $$\;\underset{{\alpha\;}_{l,i},{\alpha\;}_{e,i},{\alpha\;}_{v,i}}{{max}}{R}_{total}\;\;\;\;\;\;\;$$

i $$\;s.t.\;\;{TL}_{i,j}\le\;{T}_{th}$$

ii $$\;{\alpha\;}_{l,i},{\alpha\;}_{e,i},{\alpha\;}_{v,i}\in\;\left\{\text{0,1}\right\}$$

iii $$\;{\alpha\;}_{e,i}\;{r}_{m,n}^{ul}>{R}_{th,m}^{ul}$$

iv $$\;{{\alpha\;}_{e,i}r}_{m,n}^{dl}>{R}_{th,m}^{dl}$$

v $$\;{\alpha\;}_{l,i}\;{L}_{m,ul}\le\;{T}_{th,m}^{uRRLC,ul}$$

vi $$\;{\alpha\;}_{l,i}\;{L}_{m,dl}\le\;{T}_{th,m}^{uRRLC,dl}$$

vii $$\;{\theta\;}_{m,n}^{ul}+{\theta\;}_{m,n}^{dl}\le\;{N}_{VR}$$

Constraint (i) is guaranteed E2E latency between for link $\;{l}_{i,j}$ not exceed a certain threshold $\;{T}_{th}$ that ensures VR performance is in virtual environments. The sum of assigned RBs for each VR user must be in the range of assigned RBs for each slice in BS n, this is assured in constraints (iii) and (iv). Although, constraints (v) and (vi) ensure that latency subjected by user $\;{m}_{n}$ in uRRLC slice for uplink and downlink doesn’t exceed certain thresholds $\;{T}_{th,m}^{uRRLC,ul}$, $\;{T}_{th,m}^{uRRLC,dl}$, respectively. Additionally, constraint (vii) limits the combined number of VR sessions in uplink and downlink to not exceed total number of sessions $\;{N}_{VR}$ which this ensures that resource allocation does not exceed the system’s capacity to handle VR traffic.

### DRL based resources selection

**Figure 2:**
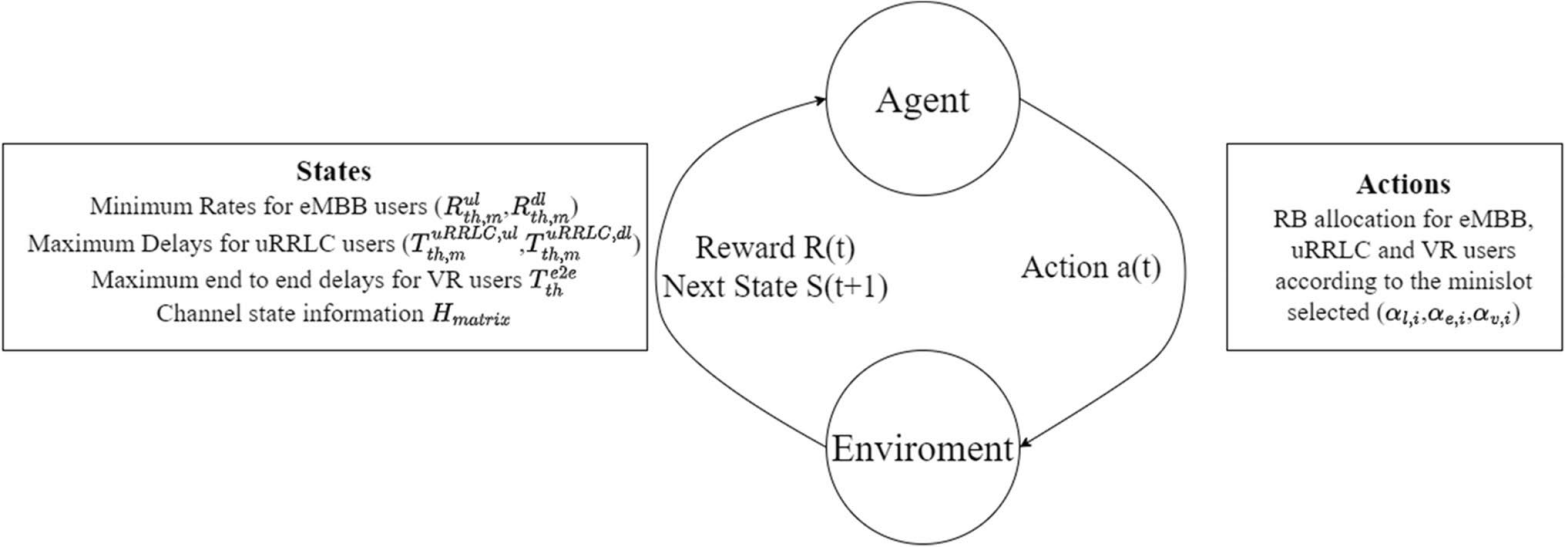
DRL orchestration.

This section explains how the proposed resource scheduling scheme works. The scheme uses dynamic resource scheduling policies based on DRL to generate resource allocation decisions. We first describe the learning framework and policy network, and then explain the design of the training algorithm. The learning-based slicing scheduling scheme uses DRL to dynamically adjust resource allocation. The state representation, action space, and reward function are designed as shown in Fig. 2. To generate efficient scheduling decisions, it is crucial for the DRL agent to understand and observe the slicing network system. To achieve this, we first design the state representation that serves as the input to the scheme. The states of the current time step slicing system observed by the agent are represented as whole E2E link properties.

Q-Learning is a well-known reinforcement learning technique that aims to find the optimal strategy through interactions with the environment that result in the highest accumulated reward 1. It is a model-free, value-based, off-policy algorithm that finds the best series of actions based on the agent’s current state. The “Q” in Q-Learning stands for quality, which represents how valuable the action is in maximizing future rewards. However, Q-Learning has some drawbacks. One of the main drawbacks is its slow convergence rate due to its iterative nature and the fact that it does not rely on any prior information when it encounters a new state. For NS, the number of possible states and actions renders a simple Q-learning approach infeasible. To overcome these limitations, DRL is used. DRL uses a Deep Neural Network (DNN) to approximate the state (s) – action (a) value function $\;q\left(s,a,\varnothing\;\right)\approx\;{Q}^{\ast}(s,a)$ where the vector contains the weights of the DNN. This DNN is trained to minimize the prediction error given by its loss function, where the target output of the DNN is defined by the optimal Q-value. By using DRL, the agent can learn from its experiences and make better decisions based on the environment. When the Q-function is modeled with the help of neural networks, it is called Deep Q-Networks (DQN). To further improve DQN for the specific context of cellular networks with diverse slices, some techniques are chosen to use in the approach. These additional techniques are crucial to use DQN for NS.

Firstly, Q-Learning is modelled as in the following steps:

- When the agent is in state s, it chooses an action a according to a policy called epsilon-greedy. In this policy, the agent chooses the action with the largest Q-value $\;Q(s,a)$ with a probability $\epsilon$, and equally chooses the other actions with a probability of $\;(1-\epsilon)/\left(\right|A|-1)$, where $\;\left|A\right|$ denotes the size of the action space.
- After the agent chooses an action, it learns the reward $\;R(s,\;a)$ from the environment, and the state transitions to the next state s’.
- Then the agent updates the Q-value function as:

7 $$\;Q(s,a)=Q(s,a)+\alpha\;\;\left(R\right(s,a)+\gamma\;\;\;max\top(a^{\prime\;}\;)Q(s^{\prime\;},a^{\prime\;}\;)-Q(s,a\left)\right)$$

Besides, deep neural network has made novel progress in the following aspects:

- Experience Replay is a technique used in reinforcement learning where the agent stores the past experience (i.e., the tuple $\;{e}_{t}=\langle{s}_{t},{a}_{t},{s{\prime\;}}_{t},\;R({s}_{t},{a}_{t})\rangle$) at episode t into a dataset $\;{D}_{t}\;=\;\{{e}_{1},\ldots,\;{e}_{t}\}$ and uniformly selects some (mini-batch) items from the dataset to update the Q-value neural network $\;Q\left(s,a\right)$.
- Network Cloning is a technique used in reinforcement learning where the agent uses a separate network Q to guide how to select an action a in state s, and the network Q is replaced by Q every C episodes.

Both experience replay and network cloning motivate to choose the off-policy Q-learning algorithm. This is because the sampling policy is only contingent on previously trained Q-value neural networks, and the updating policy, which relies on the information from the new episodes, is irrespective of the sampling policy. On the other hand, the DQL agent can collect the information (i.e., state-action-reward pair) and train its policy in the background. The learned policy is stored in the neural networks and can be conveniently transferred among similar scenarios. In other words, the DQL can efficiently perform and timely make the resource allocation decision according to its already learned policy. Before diving into the details of our proposed approach as shown in Fig. 2, we start by looking at each resource slicing problem as a Markov Decision Process (MDP). Let’s focus on a specific scenario communication resource slicing, imagine we have a bunch of gNodeB (gNB) agents. Those are like smart communication hubs observing their surroundings. These gNB agents need to allocate radio resources to the serving devices (URLLC, eMBB or VR users). For each gNB, we treat this resource allocation as a single agent MDP. In other words, each gNB agent is making decisions based on what it observes aiming to optimize resource usage for its connected devices so in a nutshell we’re using this MDP framework to tackle the resource slicing puzzle in 6G communication networks.

The State Space: The state space of a gNB-agent encompasses several key elements:

- The set of associated end-devices.
- Available RBs.
- Channel gain between the gNB and its associated devices.
- The maximum delay threshold required by the URLLC service.
- The minimum throughput for eMBB users.
- E2E link requirements for VR users, including E2E delay and the minimum transmission rate for both uplink and downlink users.

The Action Space: When it comes to the Action Space, a gNB-agent faces the task of determining which RBs should be allocated to each of the associated devices. Given that an end-device may require multiple RBs to achieve the desired QoS, we define an action as a row vector. Each element within this vector corresponds to a specific RB-device pairing.

The Reward Function: In the context of the reward function, the gNB agent’s reward hinges on its ability to allocate the necessary RBs successfully. An action is deemed successful if it adheres to the constraints set by the RB allocation model in (8). Our primary objective is to minimize E2E communication delay for VR users, so the received reward is inversely proportional to the sum of all communication delays associated by the VR users. Conversely, if the gNB-agent fails to meet these criteria, it incurs a negative reward.

## Results and discussion

To evaluate the proposed model, we conducted extensive simulations and compared it to other RL-based slicing models. The simulation environment shows the distribution of RBs across RAN slices. Each slice serves either enhanced eMBB, URLLC, or VR slice, has its traffic model and SLA. Defining SLA constraints for each slice is crucial for our problem formulation to manage RBs effectively. Area of 600 m × 150 m is considered to distribute users randomly. Some assumptions include uplink power $\;{p}_{n,ul}$ and downlink power $\;{p}_{n,dl}$​ are set at 7 dBm and 23 dBm, respectively. Additionally, bandwidth W is 1 MHz, with $\;{\sigma\;}^{2}$ at -174 dBm. File sizes for uplink $\;{L}_{m,ul}$ and downlink $\;{L}_{m,dl}$ are ranged randomly from 1 Byte to 20 Mega Byte. The network’s structure for VR slice varies based on the number of available VR users with results showcased according to the available VR users’ links. To simplify, a VR-VR link is considered whereas one VR user is transmitting and the other is receiving. The network scenario considered for the simulation results is 1 eMBB, 2 uRLLC, 2 VR slices. Moreover, the radio resources R among the RAN slices is using TTI length of 0.5 ms and sub-carrier spacing of 30 MHz for each subframe. This radio resource allocation is updated every 50 radio subframes. Therefore, the decision between resource allocation and the other is 25 ms. We compare our results with different slicing algorithms using DQN in^21^ (named in results DQN_rel) and Normalized Advantage Function (NAF)^22^ (named in results NAF_rel) which is an algorithm extend to DQN to continuous space. Additionally, DQN and NAF was running on Python 3.8 [https://www.python.org/downloads/release/python-380/] as well as using stable-baseline [https://stable-baselines3.readthedocs.io/en/master/modules/dqn.html] for DQN implementation and Keras-RL [https://github.com/keras-rl/keras-rl] for NAF implementation.

For each algorithm in every scenario, 20 simulation runs is conducted. In each comprising two consecutive phases: learning phase and testing phase. The learning phase spans 4000 decision stages which is equivalent to 70 min of simulation time. During this phase, algorithms learn from system interactions without any aforementioned knowledge of the system’s reaction. This phase is parallelizing the launch of new network slices or updating existing SLA slices. Evaluating this phase is decisive as our proposal aims to learn how is the effect of VR slice on the network operation whereas it can minimize any negative effects on service providing. Subsequently, lasting 1000 steps or 8.3 min of simulated time during the testing phase, RL algorithms stop learning and simply utilize policies acquired during the learning phase to allocate RBs at each stage.

**Figure 3:**
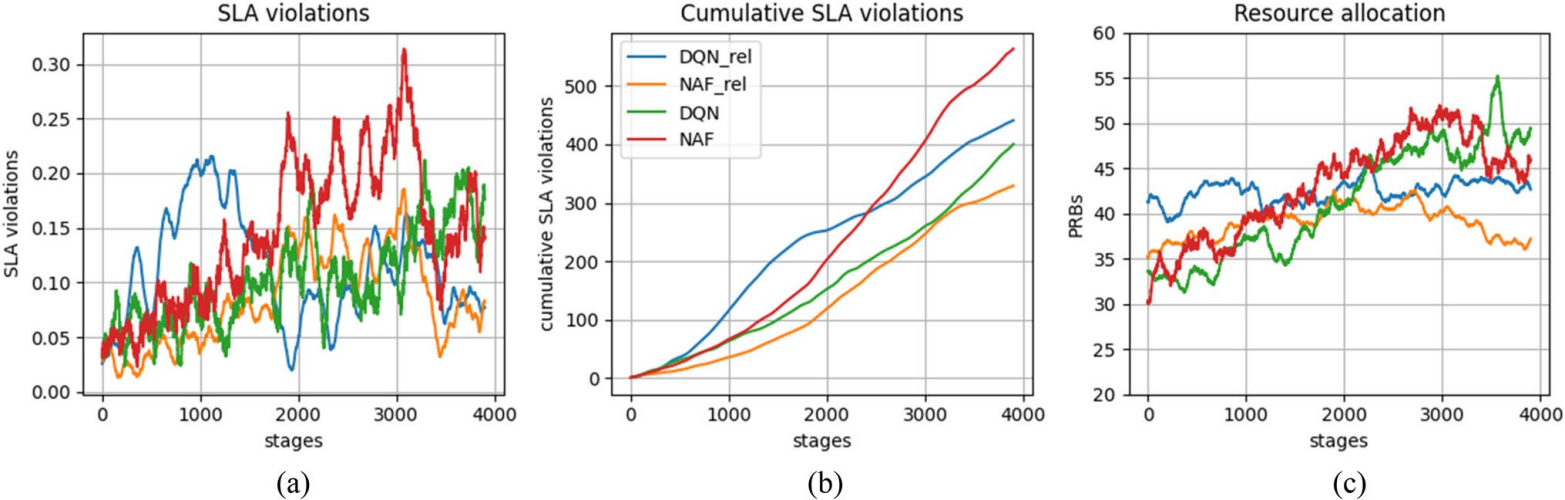
DRL learning phase performance.

In Fig. 3, the performance of NS using DQN and NAF are monitored by SLA violations that measures how is the system fails to meet SLA requirements, cumulative SLA violations that attained by combining the previous SLA violations and amount of RBs used in each slice that ranged from 0 to maximum number of RBs in each subframe. In Fig. 3 (a), it can be found that SLA violations for our proposed model using DQN or NAF is smaller than those in DQN_rel in early stages. Although, evaluating using NAF increased in middle stages this is due to more learning steps addicted. In last stages, learning for NAF relaxed as they reach it’s best solution. In contrary, DQN is almost the same during all stages. In Fig. 1(b), It can be concluded that cumulative SLA violations for our proposed model using DQN is much less than DQN_rel. This is because SLA violations for DQN almost the same during learning phase but in DQN_rel the SLA can’t be reached in early stages. Although NAF_rel it outperforms out proposed model using NAF. This means to avoid SLA violations for our proposed model, DQN is the best solution especially not all these stages needed for running. Keep in mind that the NAF algorithm requires approximating the discrete action space as a continuous one, which could impact its performance. Figure 3(c) depicts how resources are used, comparing the proposed model with DQN_re­l and NAF_rel. The purpose of this figure is to show the system’s spectral efficiency. At early stages, the proposed model shows better spectral efficiency, but as learning becomes more trained, resource allocation distinctly increases in later stages of learning. Moreover, it can be deduced that DQN generally offers a better solution in our context compared to NAF. In comparison to other research, a similar level of efficiency is observed at middle stages, but our proposed solution obtains significantly more resources after deep learning, which in turn affects the potential data rates, as shown in the next figure.

**Figure 4:**
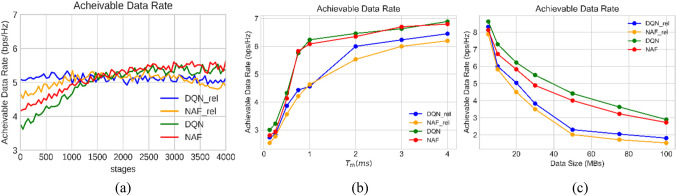
Network performance.

In Fig. 4 (a), the achievable data rate is measured with the number of learning stages increases. Early on, the achievable data rate is significantly lower compared to the performance of DQN and NAF (as used in references^21^ and ^22^, respectively). This discrepancy arises due to congestion during the initial learning stages when resources are utilized in our proposed model. However, in later learning stages, DQN handles congestion more effectively than NAF and other techniques. The system stabilizes, distributing the necessary resources to all connected users across BSs. Moreover, as can be observed, the DQN_rel has a near constant achievable data rate from the onset, suggesting that the agents are well trained to immediately make the best choices once the system is deployed, while the DQN starts off with lower achievable data rates, but gradually improve with time as learning takes place due to the many parameters addressed in the proposed model. This first drop for DQN could be due to the early irrationality in the assets allocation that the model correct as it grows and know how to prevent congestion. Whereas, DQN_rel and NAF_rel appear rather stable, one might assume so due to certain prerequisites that these models are trained to solve, for example, excluding latency issue affecting of users. However, these models do not consider latency for the VR users and it arises in our proposed model soon than the earlier models. That is, the proposed approach addresses these latency requirements better than the prior work, and increases the data rate gradually as learning progresses until the performance converges to a stable state. Additionally, we analyze the achievable data rate against changes in the time threshold for VR links which depicted in Fig. 4 (b). Increasing the time threshold alleviates congestion in selecting the appropriate number of RBs to achieve low latency. Consequently, the network becomes more relaxed in choosing the number of RBs for each VR user, resulting in a relatively stable achievable data rate starting from 3 ms for our proposed model. On the other side, it can be seen that our model is outperform other two models in all cases of time threshold adjustment. Furthermore, in Fig. 4 (c), we consider another metric: the data size sent or received by VR users. As data size increases, network congestion growths because it must accommodate larger data transfers. Consequently, the achievable data rate decreases. Conversely, at smaller data sizes, higher achievable data rates are observed. Additionally, it can be seen that when the size of data reached about 50 MBs, our proposed model is outperforming the other models; DQN_rel and NAF_rel by about 45%.

**Figure 5:**
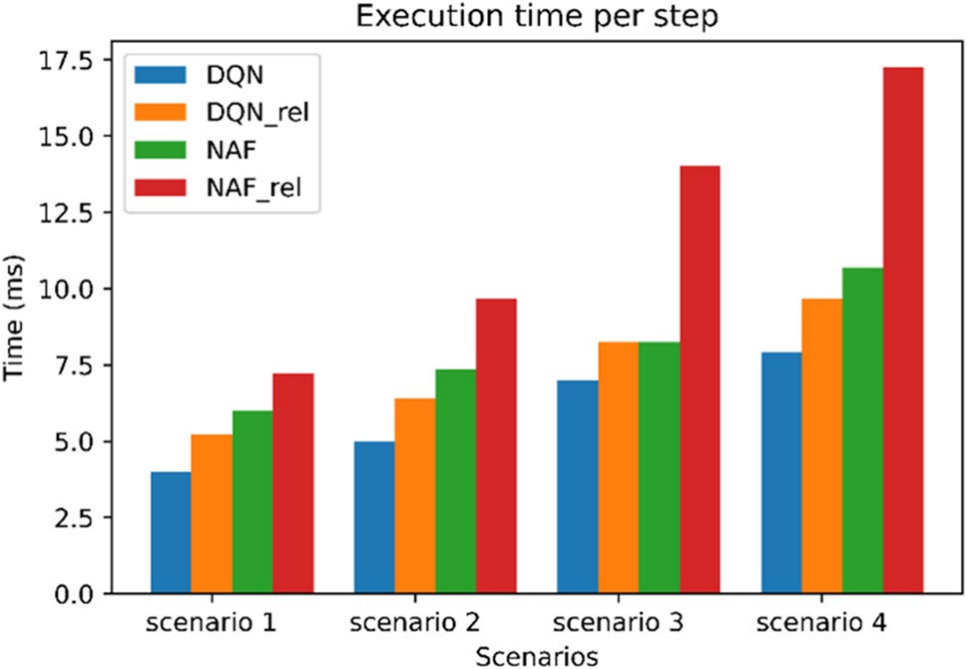
Execution time per step.

Figure 5 indicates the scalability of network performance in a large network, as it displays the execution time taken by increase number of slices. Four scenarios was created by increase 1 VR slice in each scenario. The running environment specifications used is intel core I7 and 32 GB RAM. The figure shows that the more users become online, the execution time become higher. But even this time is reasonable for the proposed model by using DQN this is because compoutational complexity is well managed by a more efficient action selection mechanism designed for the high-dimensional action spaces. Also, it can be seen that the time of NAF_rel to execute is much higher in all scenarios due to implementation differences (NAF algorithm uses the Keras-RL implementation instead of Stable Baselines). Finally, it can be concluded that the execution time increased from scenatio to scenario 4 for each algorithm by percentage as follows: DQN is 78%, NAF 98%, DQN_rel is 85% and NAF_rel is 138%. This means that the best scenario can be used for the proposed model in terms of execution time is DQN.

## Conclusion

In summary, this work directs critical needs for VR data transmission through 6G cellular networks. A VR-based 6G networks was modelled using emerging technologies like SDN and resource slicing. Thanks for SDN that offers this centralized model makes communication between VR users more organized. By integrating DRL, network RBs are balanced dynamically whereas maximizing data transmission and minimizing latency are issued. Resource management is also enhanced for slicing services such as eMBB, uRLLC and the proposed slice for VR users. Our results make comparisons that show DRL approaches achieve better results and differences compared to some previous solutions. Overall, this work contributes to effective and streamlined resource administration, essential for transmitting all VR data through 6G networks.

## Data Availability

All data supporting the findings of this study are available within the paper and its Supplementary Information. Along with original reference describing the VR communication used in this study, all used data are described in results and discussion section. Papers inspiring the work (knowing that they are included in the work) are: doi: 10.1109/LCOMM.2019.2922961, doi: 10.1109/ACCESS.2018.2881964, and our research previous part: doi: 10.1109/ACCESS.2023.3326842.

## References

[1] Liao, S., Wu, J., Li, J. & Konstantin, K. Information-centric massive IoT-Based ubiquitous connected VR/AR in 6G: a proposed caching Consensus Approach. *IEEE Internet Things J. ***8** (7), 5172–5184 (2021).

[2] Qu, H., Wang, S., Zhao, J. & Mao, L. "Inter-slice Resource Dynamic Allocation Algorithm for B5G Services," *2023 5th International Conference on **Communications, Information System and Computer Engineering (CISCE)*, Guangzhou, 46–52. 10.1109/CISCE58541.2023.10142485 (2023).

[3] Zhang, Y., Zhao, L., Liang, K., Zheng, G. & Chen, K. C. Energy Efficiency and Delay optimization of virtual slicing of Fog Radio Access Network. *IEEE Internet Things J. ***10** (3), 2297–2313 (2023).

[4] Cao, H., Kumar, N., Yang, L., Guizani, M. & Yu, F. R. Resource Orchestration and Allocation of E2E slices in Softwarized UAVs-Assisted 6G terrestrial networks. *IEEE Trans. Netw. Serv. Manage. ***21** (1), 1032–1047 (2024).

[5] Yang, P. et al. RAN slicing for massive IoT and Bursty URLLC Service Multiplexing: analysis and optimization. *IEEE Internet Things J. ***8** (18), 14258–14275 (2021).

[6] Hashima, S., Fouda, M. M., Hatano, K. & Takimoto E. "Advanced Learning Schemes for Metaverse Applications in B5G/6G Networks," *2023 IEEE **International Conference on Metaverse Computing, Networking and Applications (MetaCom)*, Kyoto, 799–804. 10.1109/MetaCom57706.2023.00150 (2023).

[7] Hieu, N. Q., Nguyen, D. N., Hoang, D. T. & Dutkiewicz, E. When virtual reality meets rate splitting multiple Access: a Joint Communication and Computation Approach. *IEEE J. Sel. Areas Commun. ***41** (5), 1536–1548 (2023).

[8] Li, Z., Zhang, H., Li, X., Ji, H. & Leung, V. C. M. "Distributed Task Scheduling for MEC-Assisted Virtual Reality: A Fully-Cooperative Multiagent Perspective," in* IEEE Transactions on Vehicular Technology. ***73** (7), 10572–10586. 10.1109/TVT.2024.3365476 (2024).

[9] Carrascal, D., Rojas, E., Lopez-Pajares D., Manso, N. & Gutierrez, E. "A scalable SDN in-band control protocol for IoT networks in 6G environments," *2023 **6th International Conference on Advanced Communication Technologies and Networking (CommNet)*, Rabat, 1–7. 10.1109/CommNet60167.2023.10365255 (2023).

[10] Nouruzi, A. et al. "Smart Resource Allocation Model via Artificial Intelligence in Software Defined 6G Networks," *ICC 2023 - IEEE International Conference on Communications*, Rome, 5141–5146. 10.1109/ICC45041.2023.10279230 (2023).

[11] Huang, J. Opportunistic capacity based resource allocation for 6G wireless systems with network slicing. *Future Generation Comput. Syst. ***140**, 390–401 (2023).

[12] Hossain, M. A. et al. AI-Assisted E2E Network Slicing for Integrated Sensing and communication in 6G networks. *IEEE Internet Things J. ***11** (6), 10627–10634 (2024).

[13] Mukherjee, A., Dey, N. & Mondal, A. iSocialDrone: QoS aware MQTT middleware for social internet of drone things in 6G-SDN slice. *Soft. Comput. ***27** (8), 5119–5135 (2023). DeR. G.Crespo.

[14] Sun, G. et al. Holistic Roadmap of trends in Radio Access Network Slicing: a Survey. *IEEE Commun. Mag. ***61** (12), 118–124 (2023).

[15] Ravi, D. A., Shah, V. K., Li, C., Hou, Y. T. & Reed, J. H. RAN slicing in Multi-MVNO Environment under Dynamic Channel conditions. *IEEE Internet Things J. ***9** (6), 4748–4757 (2022).

[16] Abdellatif, A. A. et al. Intelligent-Slicing: an AI-Assisted Network Slicing Framework for 5G-and-Beyond Networks. *IEEE Trans. Netw. Serv. Manage. ***20** (2), 1024–1039 (2023).

[17] Nguyen, T. N., Le, T. V., Nguyen, M. V., Nguyen, H. N. & Vu, S. Optimizing Resource Allocation and VNF Embedding in RAN Slicing. *IEEE Trans. Netw. Serv. Manage. ***21** (2), 2187–2199 (2024).

[18] Chiang, Y., Hsu, C. H., Chen, G. H. & Wei, H. Y. Deep Q-Learning-based Dynamic Network Slicing and Task Offloading in Edge Network. *IEEE Trans. Netw. Serv. Manage. ***20** (1), 369–384 (2023).

[19] Saad, J., Khawam, K., Yassin, M. & Costanzo, S. A three-level slicing algorithm in a multi-slice multi-numerology context. *Comput. Commun. ***212**, 324–341 (2023).

[20] Chang, W. T. & Chang, B. J. Dynamic extended sigmoid-based scheduling with virtualized RB allocation for maximizing frequency numerology efficiency in 5G/B5G NR networks. *Comput. Netw. ***237**, 110063 (2023).

[21] Li, R. Deep reinforcement learning for Resource Management in Network Slicing. *IEEE Access. ***6**, 74429–74441 (2018).

[22] Qi, C., Hua, Y., Li, R., Zhao, Z. & Zhang, H. Deep reinforcement learning with Discrete normalized advantage functions for Resource Management in Network Slicing. *IEEE Commun. Lett. ***23** (8), 1337–1341 (2019).

